# P-1387. Distribution and clinical significance of quantitative interferon-gamma release assay among elderly patients with active tuberculosis in Japan

**DOI:** 10.1093/ofid/ofaf695.1574

**Published:** 2026-01-11

**Authors:** Yoshikazu Mutoh, Yusuke Minato, Takumi Umemura, Jun Fukihara, Hajime Sasano, Kensuke Kataoka, Tomoki Kimura, Yohei Doi

**Affiliations:** Fujita Health University, Seto, Aichi, Japan; Fujita Health University, Seto, Aichi, Japan; Tosei General Hospital, Seto, Aichi, Japan; Tosei General Hospital, Seto, Aichi, Japan; Tosei General Hospital, Seto, Aichi, Japan; Tosei General Hospital, Seto, Aichi, Japan; Tosei General Hospital, Seto, Aichi, Japan; Fujita Health University, Seto, Aichi, Japan

## Abstract

**Background:**

Interferon-gamma release assays (IGRAs) are widely used for early diagnosis against TB. However, data on their diagnostic utility and clinical significance in elderly patients with active TB remian limited.

**Figure:**
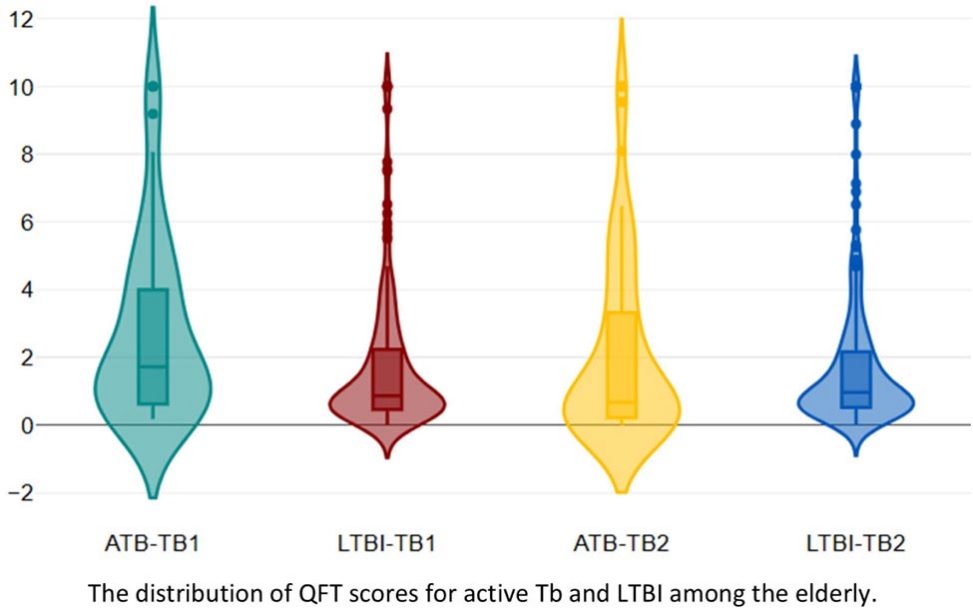

**Figure:**
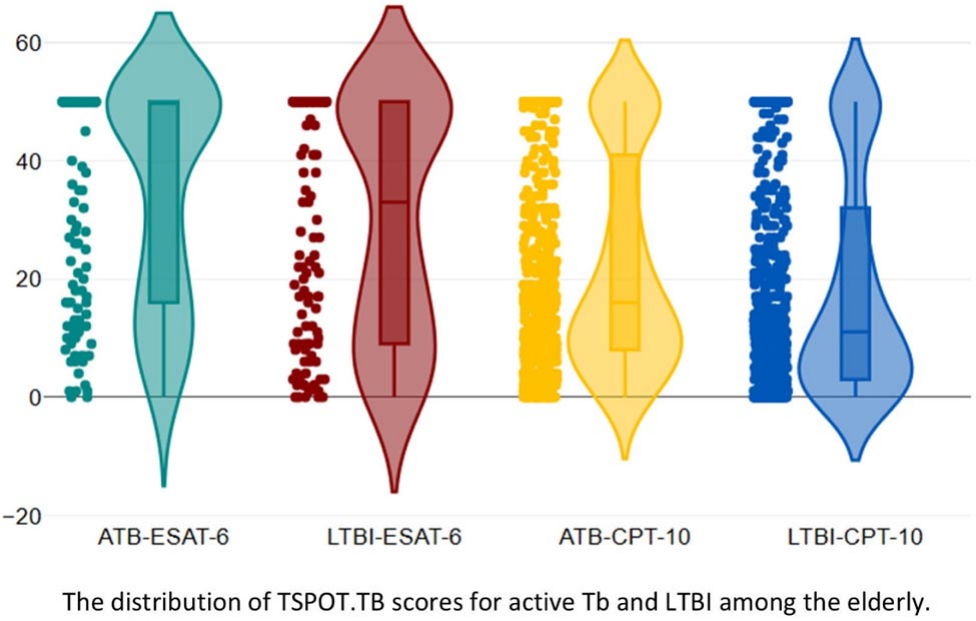

**Methods:**

We retrospectively analyzed patients over 65 years of age who underwent IGRA testing using either T-SPOT.TB (Oxford Immunotec, UK) or QuantiFERON (QFT) (Qiagen, Germany) between 2015–2024. Active pulmonary TB (ATB) was defined as TB confirmed by isolation from respiratory specimens within 90 days of IGRA testing. Latent TB infection (LTBI) was defined as IGRA-positive individuals without microbiological confirmation of TB. For individuals tested multiple times, only the most recent result was included. Values exceeding the detection threshold were recorded as maximum or minimum values.

**Results:**

A total of 12,872 T-SPOT.Tb and 7,580 QFT tests were performed during the study period, of which 3,990 and 2,620 tests, respectively, met inclusion criteria.

For T-SPOT.TB, 138 (3.5%) were diagnosed with ATB, with a positive rate of 83.3%. The median quantitative scores were 50 (IQR: 16 - 50) for ESAT-6 and 21 (IQR: 9 - 50) for CFP-10. LTBI was identified in 605 patients (15.7%), with median quantitative scores 16 (IQR: 8 - 41) for ESAT-6 and 11 (IQR: 3 - 32) for CFP-10.

For QFT, 87 (3.3%) were diagnosed with ATB, with a positive rate of 67.8%. The median quantitative scores were 1.64 (IQR: 0.62 – 4.0) for TB1 and 2.08 (IQR: 0.65 – 4.86) for TB2. The number of LTBI was 198 (7.8 %) with median quantitative scores of 0.85 (IQR: 0.46 – 2.16) for TB1 and 0.94 (IQR: 0.52 – 2.14) for TB2, respectively.

The best cut-off values were 3.1 (sensitivity: 23.0%; specificity: 87.4%), 3.6 (sensitivity: 28.7%; specificity: 85.4%) for TB1 and Tb2, 50 (sensitivity: 78.7%; specificity: 42.0%), 33 (sensitivity: 75.5%; specificity: 42.0%) for ESAT-6 and CFP-10, respectively.

**Conclusion:**

Quantitative IGRA values were higher in patients with active TB than in those with latent infection. Notably, T.SPOT-TB might be possible to differentiate ATB and LTBI in elderly populations. These findings support the potential clinical utility of TB-SPOT.TB for TB diagnosis in aging populations in Japan.

**Disclosures:**

Yoshikazu Mutoh, graduate student, MSD,　Co LTD: Honoraria Yusuke Minato, Ph.D., Shionogi & Co., Ltd.: Grant/Research Support Yohei Doi, MD, PhD, GSK: Advisor/Consultant|Meiji Seika Pharma: Advisor/Consultant|Shionogi: Advisor/Consultant|Shionogi: Honoraria

